# Cardiac shock-wave therapy in the treatment of coronary artery disease: systematic review and meta-analysis

**DOI:** 10.1186/s12947-017-0102-y

**Published:** 2017-04-12

**Authors:** Greta Burneikaitė, Evgeny Shkolnik, Jelena Čelutkienė, Gitana Zuozienė, Irena Butkuvienė, Birutė Petrauskienė, Pranas Šerpytis, Aleksandras Laucevičius, Amir Lerman

**Affiliations:** 1 Clinic of Cardiac and Vascular Diseases, Faculty of Medicine, Vilnius University, Vilnius, Lithuania; 2 Centre of Cardiology and Angiology, Vilnius University Hospital Santariskiu Klinikos, Vilnius, Lithuania; 3 Moscow State University of Medicine and Dentistry, Moscow, Russia; 4 Yale- New Haven Health Bridgeport Hospital, Connecticut, United States of America; 5 Centre of Innovative Medicine, Vilnius, Lithuania; 6 Division of Cardiovascular Diseases, Mayo Clinic, Rochester, Minnesota United States of America; 7 Room No A311, Santariskiu str. 2, 08661 Vilnius, Lithuania

**Keywords:** Cardiac shock wave therapy, coronary artery disease, stable angina pectoris, refractory angina

## Abstract

**Aim:**

To systematically review currently available cardiac shock-wave therapy (CSWT) studies in humans and perform meta-analysis regarding anti-anginal efficacy of CSWT.

**Methods:**

The Cochrane Controlled Trials Register, Medline, Medscape, Research Gate, Science Direct, and Web of Science databases were explored. In total 39 studies evaluating the efficacy of CSWT in patients with stable angina were identified including single arm, non- and randomized trials. Information on study design, subject’s characteristics, clinical data and endpoints were obtained. Assessment of publication risk of bias was performed and heterogeneity across the studies was calculated by using random effects model.

**Results:**

Totally, 1189 patients were included in 39 reviewed studies, with 1006 patients treated with CSWT. The largest patient sample of single arm study consisted of 111 patients. All selected studies demonstrated significant improvement in subjective measures of angina symptoms and/or quality of life, in the majority of studies left ventricular function and myocardial perfusion improved. In 12 controlled studies with 483 patients included (183 controls) angina class, Seattle Angina Questionnaire (SAQ) score, nitrates consumption were significantly improved after the treatment.

In 593 participants across 22 studies the exercise capacity was significantly improved after CSWT, as compared with the baseline values (in meta-analysis standardized mean difference SMD = −0.74; 95% CI, −0.97 to −0.5; *p* < 0.001).

**Conclusions:**

Systematic review of CSWT studies in stable coronary artery disease (CAD) demonstrated consistent improvement of clinical variables. Meta-analysis showed a moderate improvement of exercise capacity.

Overall, CSWT is a promising non-invasive option for patients with end-stage CAD, but evidence is limited to small sample single-center studies. Multi-center adequately powered randomised double blind studies are warranted.

## Background

A substantial number of patients suffer from disabling angina despite having undergone invasive treatment methods and continuation on optimal medical treatment (OMT) [1]. Such condition is defined as a refractory angina (RFA) [2]. In many cases, stable coronary artery disease (CAD) becomes too diffuse and extensive to be treated by traditional revascularization methods. The annual mortality rate of RFA in recent studies is in the range of 3–4% [3, 4].

Several new alternative treatment methods of RFA are being investigated. A number of studies showed that transmyocardial [5] and percutaneous myocardial laser revascularization [6, 7], spinal cord stimulation [8] and stem cell therapy [9–11] may reduce angina symptoms and improve exercise capacity, myocardial perfusion and function. Nevertheless, these treatment modalities are invasive, quite expensive or still at a preclinical stage.

Enhanced external counter-pulsation is a non-invasive option suggested for CAD patients. However, the recent studies were inconclusive and found no or small differences between test and control groups with respect to change in angina or exercise duration [12, 13].

Ultrasound-guided cardiac shock wave (SW) therapy is another promising non-invasive modality in patients with stable CAD. Experimental studies showed that SW might induce shear stress to endothelial cells and produce complex cascade of short- and long-term reactions leading to angiogenesis [14, 15]. The observed immediate increase in blood flow due to local vasodilation and the formation of new capillaries in the treated tissue [16–18] has led to its application in cardiovascular medicine. Since 1999 [19], cardiac shock-wave therapy (CSWT) as a tool for the management of RFA has been investigated in a considerable number of clinical studies.

Our aim was to systematically review and analyse currently available data from CSWT studies in humans and perform meta-analysis regarding efficacy of CSWT on exercise capacity.

## Materials and methods

Inclusion criteria, search strategy, methods of data collection and analysis were elaborated in a protocol.

### Data sources

We searched for articles evaluating the efficacy of CSWT in CAD patients from the following medical bibliographic databases: Cochrane Controlled Trials Register, Medline, Medscape, Research Gate, Science Direct, Web of Science (from 1999 to April of 2016), and Google Web. Publications were selected by pre-defined criteria and reviewed by two authors (GB, ES) following PRISMA statement [20]. Disagreements were discussed with other author (JC). The search terms included coronary artery disease, ischemic heart disease, refractory angina treatment, stable angina treatment combined with extracorporeal cardiac shock wave therapy, myocardial shock wave therapy, extracorporeal myocardial revascularisation. We also searched for references in review articles and abstracts.

### Study selection criteria

In order to be included, trials had to assess the treatment with CSWT of CAD patients, written in English. Selected studies included patients with stable CAD proven by coronary angiography or computed tomography angiography, not amenable to revascularization, angina class II-IV (Canadian Cardiology Society, CCS), despite OMT, and documented stress induced myocardial ischemia. Trials investigating combination of CSWT with stem cell therapy were not included.

### Data extraction

Information on 1) study design (including study type, method of randomization and blinding of patients, study personnel and outcome assessors), 2) sample size and patients characteristics (including age, sex), 3) intervention strategies (including treatments schedule, follow up duration), 4) outcome measures (including (short-acting nitrates consumption per week, CCS angina class and New York Heart Association [NYHA] functional class, Seattle Angina Questionnaire (SAQ) scores, and parameters of the functional tests as exercise duration, workload, global and regional left ventricular [LV] function, myocardial perfusion) were extracted into Microsoft Excel (Microsoft, Seattle, Wash., USA) spread sheets.

### Statistical analysis

Variables were presented as mean value ± standard deviation (SD) for continuous data with normal distribution and as median with interquartile range (IQR: Q1, Q3) for data not normally distributed, whereas categorical variables were expressed as number (%).

Assessment of risk of bias randomized trials was performed in accordance with the Cochrane Collaboration tool [21] and was based on information on concealment of allocation and random sequence generation, blinding of participants and personnel, incomplete outcome data and selective reporting. For risk of bias assessments the low/unclear/high scale was used.

The effect sizes used in each study are presented as standardized mean difference (SMD) with 95% confidence interval (CI) to allow for combination of different measurements of exercise capacity. In line with Cohen's classification [22], effect sizes were divided into trivial (Cohen's d ≤0.2), small (<0.5), moderate (<0.8), and large (>0.8).

Heterogeneity was assessed by using the chi-square test for heterogeneity and the I^2^ statistic to determine the proportion of variation attributable to heterogeneity among studies. Values of I^2^ considered as low (<25%), moderate (25–50%) and high (>50%) heterogeneity. Meta-analysis results are presented as forest plots. Random effects model according to Der Simonian-Laird was used to verify the significant evidence of heterogeneity between the results of studies. Publication bias was estimated by drawing funnel plot. The analysis was performed using RevMan 5.3 software (Copenhagen, The Nordic Cochrane Centre) [23].

## Results

### Study characteristics and patient population

**Fig. 1:**
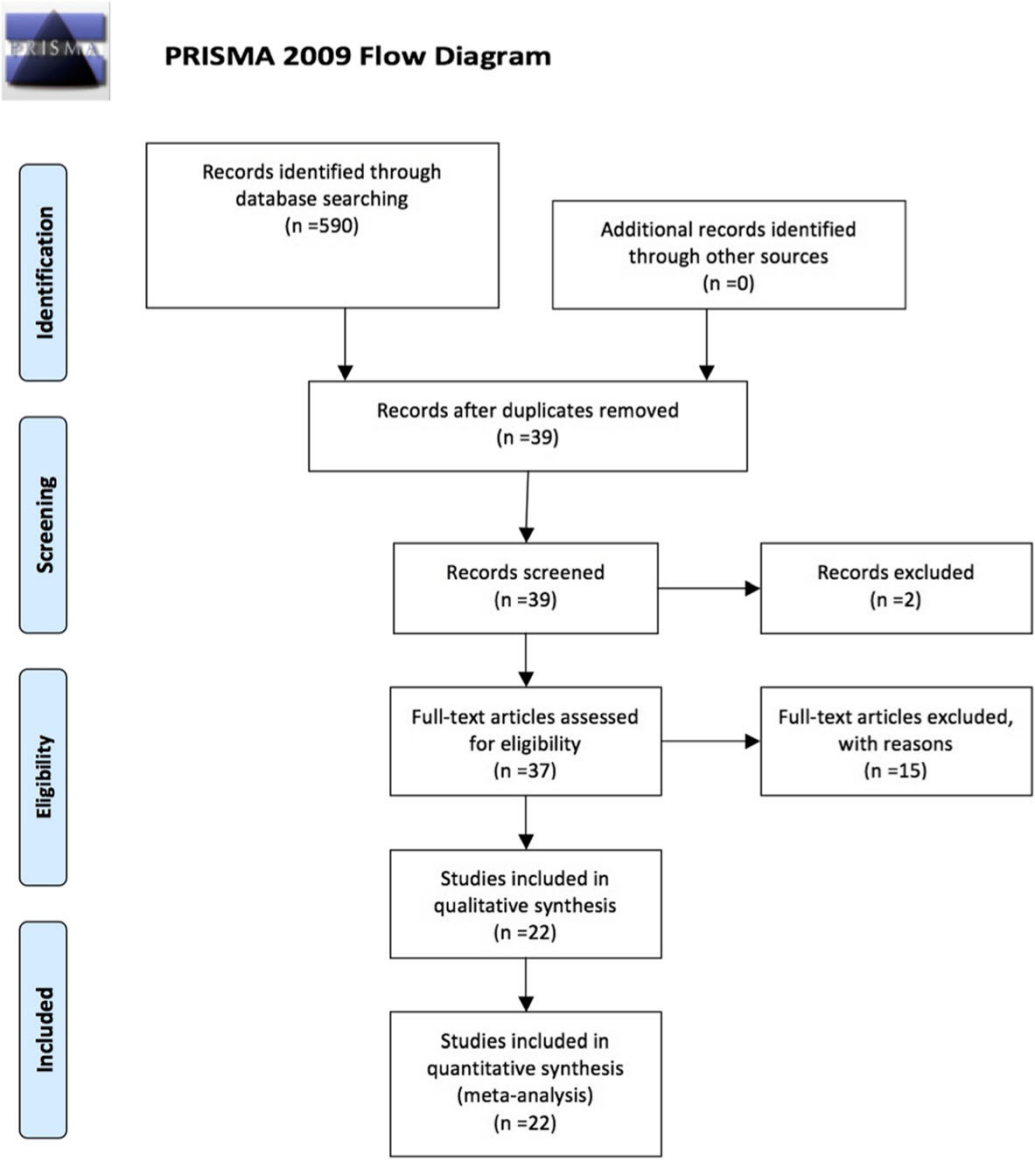
Study flow diagram

From 590 identified publications after exclusion of irrelevant, experimental, animal and non-English studies 39 studies were selected for review following the PRISMA statement [20] (Fig. 1, Table 1); their common characteristics are summarized in Table 2.

**Table 1:** PRISMA checklist

Section/topic	Number	Checklist item	Reported on page #
TITLE			
Title	1	Identify the report as a systematic review, meta-analysis, or both.	1
ABSTRACT			
Structured summary	2	Provide a structured summary including, as applicable: background; objectives; data sources; study eligibility criteria, participants, and interventions; study appraisal and synthesis methods; results; limitations; conclusions and implications of key findings; systematic review registration number.	2
INTROCUTION			
Rationale	3	Describe the rationale for the review in the context of what is already known.	3
Objectives	4	Provide an explicit statement of questions being addressed with reference to participants, interventions, comparisons, outcomes, and study design (PICOS).	3
METHODS			
Protocol and registration	5	Indicate if a review protocol exists, if and where it can be accessed (e.g., Web address), and, if available, provide registration information including registration number.	3
Eligibility criteria	6	Specify study characteristics (e.g., PICOS, length of follow-up) and report characteristics (e.g., years considered, language, publication status) used as criteria for eligibility, giving rationale.	4
Information sources	7	Describe all information sources (e.g., databases with dates of coverage, contact with study authors to identify additional studies) in the search and date last searched.	4
Search	8	Present full electronic search strategy for at least one database, including any limits used, such that it could be repeated.	4
Study selection	9	State the process for selecting studies (i.e., screening, eligibility, included in systematic review, and, if applicable, included in the meta-analysis).	4
Data collection process	10	Describe method of data extraction from reports (e.g., piloted forms, independently, in duplicate) and any processes for obtaining and confirming data from investigators.	4
Data items	11	List and define all variables for which data were sought (e.g., PICOS, funding sources) and any assumptions and simplifications made.	4
Risk of bias in individual studies	12	Describe methods used for assessing risk of bias of individual studies (including specification of whether this was done at the study or outcome level), and how this information is to be used in any data synthesis.	4–5
Summary measures	13	State the principal summary measures (e.g., risk ratio, difference in means).	Table 2, 4–5
Synthesis of results	14	Describe the methods of handling data and combining results of studies, if done, including measures of consistency (e.g., I^2^) for each meta-analysis.	4–5

**Table 2:** Common characteristics of selected human studies of cardiac shock wave therapy

Author (year)	Study population	Stress test, used to detect myocardial ischemia	Patients, Total control (n)	Age (years)	Sex, male, n (%)	Follow up, months
Non-controlled studies						
Caspari G. H. et al. (1999) [19]	Stable angina	SPECT	9/-	65 ± 7	nd	6^d^
Gutersohn A. et al. (2003) [51]	Stable angina	SPECT, ET	25/-	66 ± 7.3	nd	6^d^
Gutersohn A. et al. (2005) [52]	Stable angina	SPECT	14/-	66	nd	12^e^
Gutersohn A. et al. (2006) [53]	Stable angina	SPECT	23/-	66	nd	60^d^
Fukumoto Y.et al. (2006) [54]	Stable angina	ET, SPECT	9/-	67.8	5 (55.5%)	12^d^
Lyadov K. et al. (2006) [55]	Stable angina	DSE, CPET	13/-	59.6 ± 6.9	11 (85%)	1^e^
Naber C. et al. (2007) [56]	Stable angina	SPECT	25/-	63.8 ± 8.2	nd	3^d^
KhattabA.A. et al. (2007) [57]	Stable angina	SPECT	10/-	nd	nd	1^d^
Naber C. et al. (2008) [58]	Stable angina	SPECT	24/-	63.8 ± 8.2	18 (75%)	3^d^
Takayama T. et al. (2008) [28]	Stable angina	SPECT	17/-	67.5	17 (100%)	6^d^
Wang Y. et al. (2010) [59]	Stable angina	DSE, SPECT	9/-	63.7 ± 5.7	9 (100%)	1^d^
Faber L. et al. (2010) [60]	Stable angina	PET, CPET	16/-	66 ± 10	nd	1^d^
Vainer J. et al. (2010) [61]	Stable angina	ET, SPECT	22/-	69 ± 7	18 (81.8%)	4^d^
Vasyuk Y. A. et al. (2010) [25]	Ischemic HF	DSE, SPECT	24/-	63.3 ± 6.1	20 (83.3%)	6^d^
Alunni G. et al. (2011) [62]	Stable angina	SPECT	16/-	71 ± 5.6	12 (80%)	12
Vainer J. et al. (2012) [63]	Stable angina	SPECT	50/-	68 ± 9	40 (80%)	4^d^
Alunni G. et al. (2013) [64]	Stable angina	SPECT	25/-	nd	nd	6^d^
Gabrusenko S.A. et al. (2013) [29]	Stable angina	SPECT	17/-	67.4 ± 8.6	14 (82.4%)	1^e^
Zuoziene G. et al. (2013) [65]	Stable angina	DSE, SPECT	40/-	67.7 ± 7	30 (75%)	3^d^
Prinz C. et al. (2013) [66]	Stable angina	ET, PET	43/-	67 ± 10	nd	1^d^
Cassar A. et al. (2014) [27]	Stable angina	ET, SPECT	15/-	65.0 ± 12.1	13 (86.7)	4^d^
Faber L. et al. (2014) [67]	Stable angina	PET	47/-	67 ± 10	nd	1,5^d^
Prasad M. et al. (2015) [68]	Stable angina	SPECT, ET	111/-	62.9 ± 10.9	98 (83.7)	3–6^e^
Kaller M. et al. (2015) [49]	Stable angina	PET, ET	21/-	65 ± 10	13 (61.9%)	1.5–2^d^
Cai HY et al. (2015) [30]	Stable angina	ET	26/-	63 ± 10	23 (88.5%)	4^d^
Liu BY et al. (2015) [69]	Stable angina	SPECT	11/-	nd	nd	12^d^
Vainer J. et al. (2016) [70]	Stable angina	ET, SPECT	33/-	69.7 ± 8	27 (82%)	4^d^
Non-randomized, controlled studies						
Kikuchi Y. et al. (2010)^c^ [31]	Stable angina	CPET	8/8	70 ± 3	5 (62.5%)	3^d^
Kazmi W.H. et al. (2012) [71]	Stable angina	SPECT	86/43	57.7 ± 10.5	73 (84.5%)	6^d^
Alunni G. et al. (2014) [72]	Stable angina	SPECT	72/29	70 ± 5.3	60 (83.3%)	6^d^
Nirala S. et al. (2016) [73]	Stable angina	ET, DSE	52/11	63.4 ± 10.8	43 (82.7%)	72^d^
Randomized, controlled studies						
Peng Y.Z. et al. (2012) [26]	Ischemic HF	SPECT	50/nd	nd	nd	1^d^
Wang Y. et al. (2012)^a^ [24]	Stable angina	DSE, SPECT	55/14	64.1 ± 9.8	47 (85%)	12^e^
Zhao L. et al. (2015)^b^ [74]	Stable angina	SPECT, ET	87/27	66.8 ± 8.4	68 (78%)	12^e^
Randomized, placebo controlled studies						
Schmid J.P. et al. (2006) [75]	Stable angina	SPECT	15/8	68 ± 8	14 (60%)	3^d^
Yang P. et al. (2012)^a^ [76]	Stable angina	SPECT	45/20	67 ± 8.3	36 (80%)	3^e^
Leibowitz D. et al. (2012)^a^ [77]	Stable angina	ET, SPECT	28/10	63.3 ± 9.2	24 (85.7%)	3^d^
Schmid J.P. et al. (2013) [78]	Stable angina	CPET	21/10	68.2 ± 8.3	19 (90.5%)	3^d^
Yang P. et al. (2013)^a^ [79]	Stable angina	SPECT	25/11	65.1 ± 8.5	18 (72%)	6^d^

Continuous variables were expressed as mean value ± standard deviation (SD), whereas categorical variables were expressed as percentages

*ET* ECG Exercise test, *CPET* cardiopulmonary exercise test, *DSE* dobutamine stress echocardiography, *PET* positron emission tomography, *SPECT* single photon emission computed tomography; nd = no data; ^a^double blind; ^b^single blind; ^c^double blind, placebo controlled, crossover design; ^d^time after the end of treatment (treatment ends at 9^th^ treatment week); ^e^time from the treatment initiation

In total, 1189 patients were included with 1006 patients treated with CSWT (483 patients underwent CSWT in controlled studies), 183 patients entered control groups. The mean age of patients was 66 ± 6.7 years, 80.8% were men. Study sample size was from 8 to 111 patients; duration of follow up lasted from 1 to 72 months.

No procedure related adverse events and good treatment tolerance were reported.

Studies did not include patients with acute coronary syndromes at least 3 months before enrolment, recent revascularization and thrombus in the left ventricle.

In most studies the treatment protocol consisted of nine sessions conducted over a 9-week period with three treatment series performed on the 1^st^, 5^th^ and 9^th^ week. Shock waves were applied to targeted area of myocardial ischemia detected by imaging stress tests. Wang showed that a modified regimen of nine treatment sessions within 1 month had similar therapeutic effect, as compared to the standard treatment protocol [24]; only a standard treatment group from this study was included in meta-analysis in order to reduce possible heterogeneity.

Risk of bias and quality assessment of controlled studies is shown in Table 3.

**Table 3:** Quality and risk of bias assessment for randomized studies

	Wang Y. 2012 [24]	Zhao L. 2015 [74]	Yang P. 2012 [76]	Leibowitz D. 2012 [77]	Schmid J.P. 2013 [78]	Yang P. 2013 [79]
Random sequence generation	high risk	low risk	high risk	high risk	high risk	high risk
Allocation concealment	high risk	high risk	high risk	high risk	high risk	high risk
Blinding of participants	high risk	low risk	high risk	low risk	low risk	high risk
Blinding of personnel who provide CSWT treatment	high risk	high risk	high risk	high risk	high risk	high risk
Blinding of outcome assessment	unclear risk	high risk	high risk	high risk	high risk	high risk
Incomplete outcome data	high risk	high risk	low risk	high risk	high risk	low risk
Selective reporting	low risk	low risk	low risk	low risk	low risk	low risk
Blinding of CWST procedure	high risk	low risk	high risk	low risk	low risk	high risk
Endpoints were based on sample size calculation	high risk	high risk	high risk	high risk	high risk	high risk
Complete testing in both groups	low risk	low risk	low risk	low risk	low risk	low risk

*CSWT* cardiac shock wave therapy

### Cardiac shock wave therapy effect on clinical variables

All selected studies demonstrated positive effect of CSWT on clinical variables (results of controlled studies are shown in Table 4). In CSWT patients CCS angina scale (31 studies) and NYHA class (13 studies) have reduced by 1 (1, 1) and 1 (0, 1), respectively, compared with the baseline values. The frequency of weekly nitroglycerine use declined from 40 to 75% (in 16 related studies).

**Table 4:** Effect of cardiac shock wave therapy in human controlled studies: clinical and quality of life parameters

		Period	CCS angina class	Nitroglycerine consumption	NYHA class	Seattle angina questionnaire
P. Yang 2013 [79]	Test group (N=14)	Baseline	2.0 (1.0, 3.0)	2.0 (0.0, 3.0)	2.0 (1.0, 2.0)	73.5 (60.5, 81.0)
		Post treatment	1.0 (1.0, 2.0)*	1.0 (0.0, 2.0)	1.0 (1.0, 1.0)*	82.0 (74.5, 88.0)*
	Placebo group (N=11)	Baseline	2.0 (1.0, 3.0)	2.0 (1.0, 3.0)	1.0 (1.0, 2.0)	73.0 (63.0, 80.0)
		Post treatment	2.0 (1.0, 2.0)	2.0 (0.0, 2.0)	2.0 (1.0, 2.0)	78.0 (69.0, 85.0)
Y. Wang 2012 [24]	I group (standard treatment) (N=20)	Baseline	2 (1, 2)	1 (0, 2)	1.5 (1, 3)	64.9±11.72
		Post treatment	1 (1, 1)*	0 (0, 1)	1 (1, 1)	75.0±10.45*
	II group (modified treatment) (N=21)	Baseline	3 (2, 3)	2 (0, 3)	2 (1, 2.5)	67.9±13.0
		Post treatment	2 (1, 2)	0 (0, 1)	1 (1, 1)	76.14±12.28
	Control group (N=14)	Baseline	2 (2, 3)	1 (0, 4)	2 (1, 3)	63.21±11.89
		Post treatment	2 (1, 2.3)	0 (0, 2)	1 (1, 2.3)	60.14±12.82
P. Yang 2012 [76]	Test group (N=25)	Baseline	2.72±0.46	2.35±0.86	2.16±0.69	65.96±11.78
		Post treatment	1.46±0.58*	1.0±0.73*	1.48±0.65*	76.4±11.78*
	Placebo group (N=20)	Baseline				
		Post treatment	No significant changes	No significant changes	No significant changes	No significant changes
S. Nirala 2016 [73]	Test group (N=41)	Baseline	2.21±0.85	1.34±1.35	1.85±0.96	66.34±12.34
		Post treatment	1.14±0.57	0.21±0.82^*^	1.04±0.49**	79.92±25.14**
	Control group (N=11)	Baseline	1.81±0.75	1.36±1.62	1.36±0.67	84±7.61
		Post treatment	2.18±0.75	2±1.18	2.09±0.94	72.72±12.33
Y. Kikuchi 2010 [31]	Test group (N=8)	Baseline	3.0	4.0	-	-
		Post treatment	2.25*	1.0*	-	-
	Placebo group (N=8)	Baseline	2.75	4.0	-	-
		Post treatment	2.75	3.0*	-	-
W.H. Kazmi 2012 [71]	Test group (N=43)	Baseline	2.63±0.7	-	2.48±0.6	-
		Post treatment	1.95±0.8**	-	1.95±0.5**	-
	Control group (N=43)	Baseline	2.63±0.7	-	2.48±0.6	-
		Post treatment	2.63±0.7	-	2.46±0.6	-
G. Alunni 2014 [72]	Test group (N=43)	Baseline	2.67±0.75	26(60.5%)	2.51±0.74	-
		Post treatment	1.33±0.57**	9 (20%)*	1.23±0.42**	-
	Control group (N=29)	Baseline	2.52±0.78	18 (41%)*	2.32±0.79	-
		Post treatment	1.92±0.69	13 (44.8%)*	1.73±0.59	-

*CCS* Canadian Cardiovascular Society Angina Class, nitroglycerine consumption is expressed as number of tablets per day, *NYHA* New York Heart Association class, * = *p*<0.05 compared to baseline, ** = *p*<0.001 compared to baseline

### Meta-analysis of cardiac shock wave therapy effect on exercise capacity

Two studies investigating ischemic heart failure population were excluded from meta-analysis [25, 26].

**Fig. 2:**
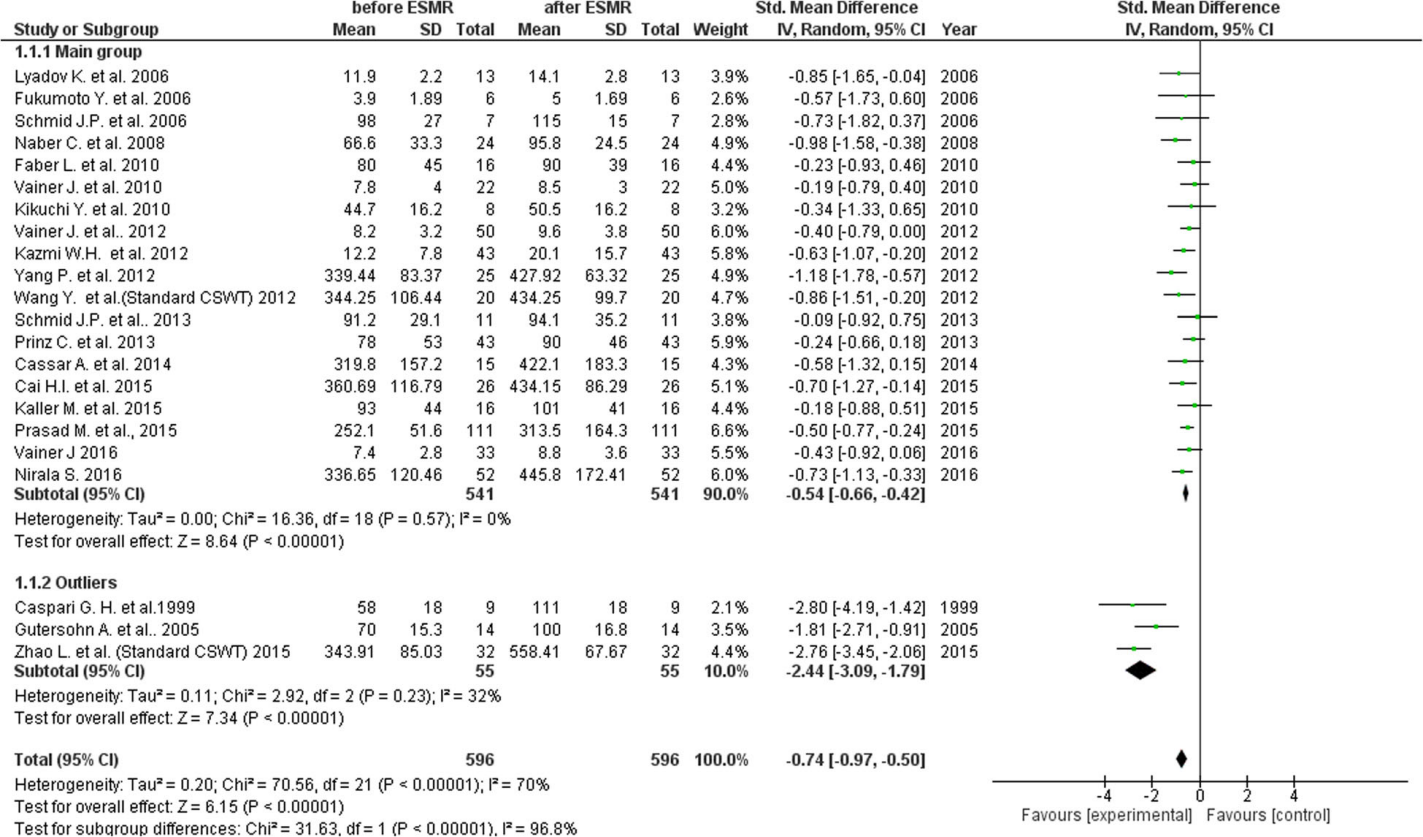
Meta-analysis of overall impact of cardiac shock wave therapy on exercise capacity

From remaining 37 studies only 22 studies provided data suitable to be included in meta-analysis to evaluate the impact of CSWT on the parameters of exercise tolerance (mean and standard deviation or standard error of mean values, both baseline and post procedure), (Fig. 2, Table 5).

**Table 5:** Effect of cardiac shock wave therapy on the parameters of exercise capacity

Study (year)	Study type	Number of patients who underwent CSWT	Value before CSWT	Value after CSWT	Measurement unit
Caspari G.H. et al. (1999) [19]	Single arm	9	58±18	111±18	Wt
Gutersohn A. et al. (2005) [52]	Single arm	14	70±15.3	100±16.8	Wt
Lyadov K. et al. (2006) [55]	Single arm	13	11.9±2.2	14.1±2.8	VO_2_ ml/kg/min
Fukumoto Y. et al. (2006) [54]	Single arm	9	3.9±1.9	5±1.7	Met
Schmid J.P. et al. (2006) [75]	Randomized, Placebo controlled	7	98±27	115±15	Wt
Naber C. et al. (2008) [58]^a^	Single arm	24	66.6±33.3	95.8±24.5	Wt
Faber L. et al. (2010) [60]	Single arm	16	80±45	90±39	Wt
Vainer J. et al. (2010) [61]	Single arm	22	7.8±4	8.5±3	Minutes
Kikuchi Y. Et al. (2010) [31]	Placebo controlled	8	44.7±16.2	50.5±16.2	Wt
Vainer J. et al. (2012) [63]	Single arm	50	8.2±3.2	9.6±3.8	Minutes
Kazmi W.H. et al. (2012) [71]	Controlled	43	12.2±7.8	20.1±15.7	Minutes
Yang P. et al. (2012) [79]	Randomized, Placebo controlled	25	339.44±83.3	427.9±63.3	Meters
Wang Y. et al. (2012) [24]^b^	Randomized, controlled	31	344.3±106.4	434.3±99.7	Meters
Schmid J.P. et al. (2013) [78]	Randomized, Placebo controlled	11	91.2±29.1	94.1±35.2	Wt
Prinz C. et al. (2013) [66]	Single arm	43	78±53	90±46	Wt
Cassar A. et al. (2014) [27]	Single arm	15	319.8±157.2	422.1±183.3	Seconds
Zhao L. et al. (2015) [74]^b^	Randomized, controlled	32	343.9±85.0	489.4±72.2	Seconds
Prasad M. et al. (2015) [68]	Single arm	111	252.1±51.6^c^	313.5±164.3	Seconds
			457.0±146.8^d^	606.0±126.4	
Kaller M. et al. (2015) [49]	Single arm	16	93±44	101±41	Wt
Cai HY. et al. (2015) [30]	Single arm	26	360.7±116.8	434.2±86.3	Meters
Nirala S. et al. (2016) [73]	Controlled	41	336.7±120.5	445.8±172.4	Meters
Vainer J. et al. (2016) [70]	Single arm	33	7.4±2.8	8.8±3.6	Minutes

All valuables presented as mean ± SD, ^a^valuable presented as mean ± SE, SE calculated into SD using standard formulas; ^b^group with standard CSWT protocol, ^c^Bruce protocol, ^d^modified Bruce protocol

Across 22 contributing studies (596 participants) the exercise capacity was significantly improved after CSWT, as compared with the baseline values (SMD = −0.74; 95% CI, −0.97 to −0.5; *p* < 0.001, I^2^ = 70%, Fig. 2); mean follow up period made 8 months (range 1–72 months).

In order to explain heterogeneity, we performed sensitivity analysis by removing from analysis one of the studies at a time. Overall effect changed to −0.61, 95% CI (−0.78 to −0.44), *p* < 0.001 when excluding study of Zhao L. et al. (2015) and to −0.77, 95% CI (−1.01 to −0.52), *p* < 0.001 when excluding study of Prinz C. et al (2013).

**Fig. 3:**
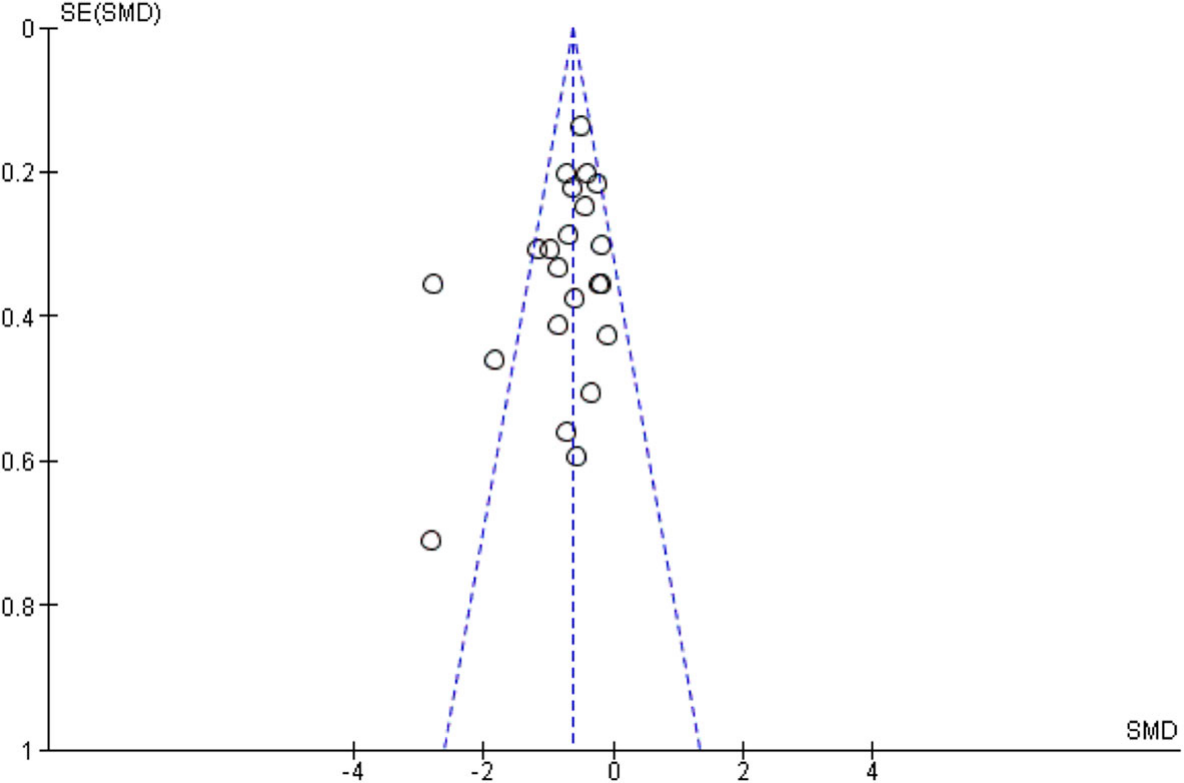
Funnel plot of the meta-analysis. The standardized mean difference (SMD) on the x-axis is plotted against the standard error (SE) of the log(SMD) on the y-axis. A symmetrical distribution of studies indicates the absence of publication bias. An asymmetrical distribution with, for example, relatively more smaller studies with a positive result (in the lower right part of the plot) would suggest the presence of publication bias

Funnel plot analysis was performed in order to evaluate publication bias (Fig. 3). The funnel plot graph was asymmetrical and three outliers were identified representing studies of Caspari, Gutersohn and Zhao group. Without these studies heterogeneity decreased to I^2^ = 0%, *p* = 0.57 with SMD = −0.54; 95% CI, −0.66 to −0.42; *p* < 0.001.

Interestingly, in uncontrolled studies treatment effect was smaller than in controlled studies (SMD -0.59 (−0.81, −0.36) vs −0.93 (−1.44, −0.42)).

### Cardiac shock wave therapy effect on left ventricular function

**Fig. 4:**
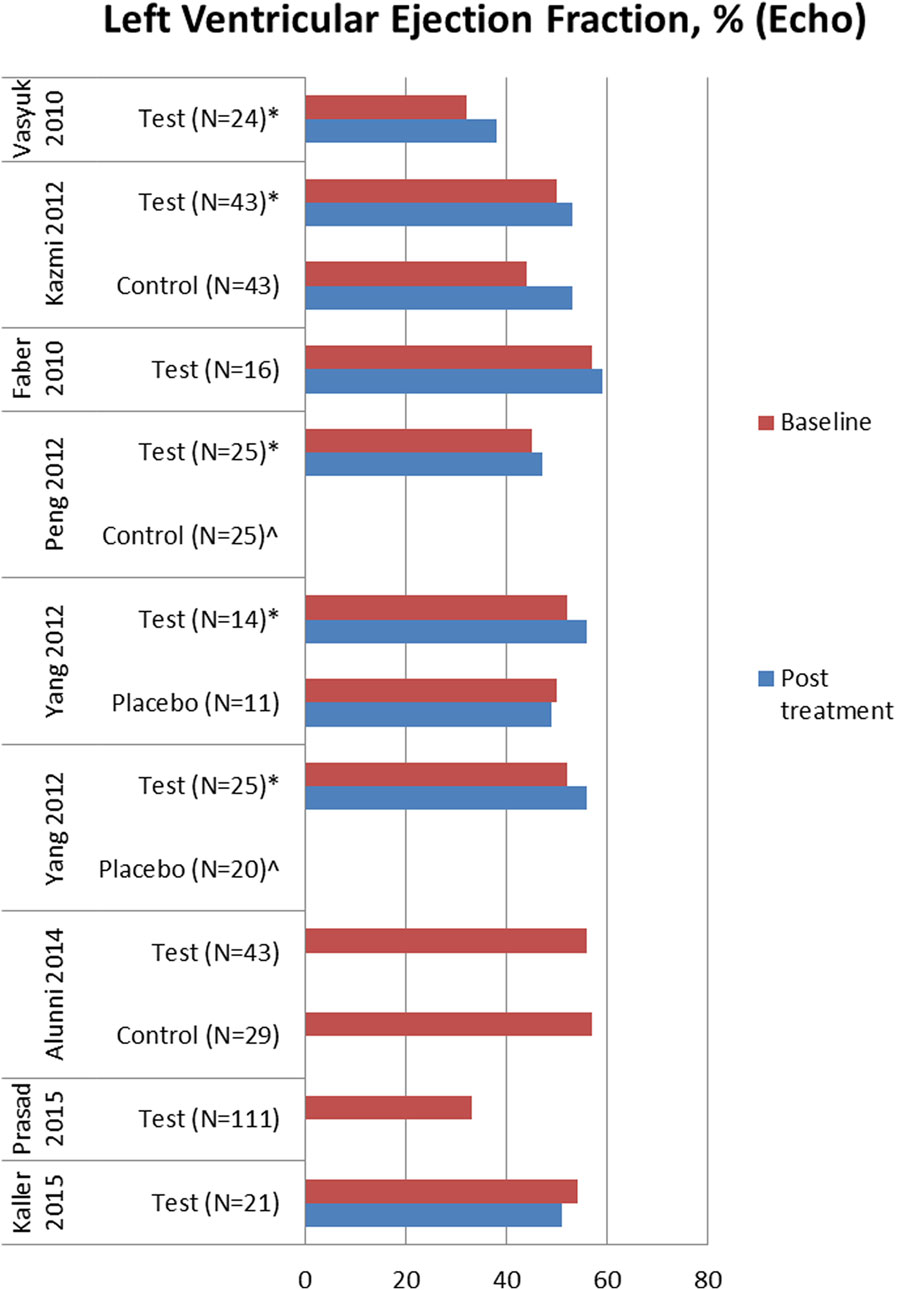
Changes of left ventricular ejection fraction evaluated by echocardiography in available cardiac shock wave therapy studies. * = *p*<0.05 compared to baseline, ^ = no significant changes, no figures indicated

**Fig. 5:**
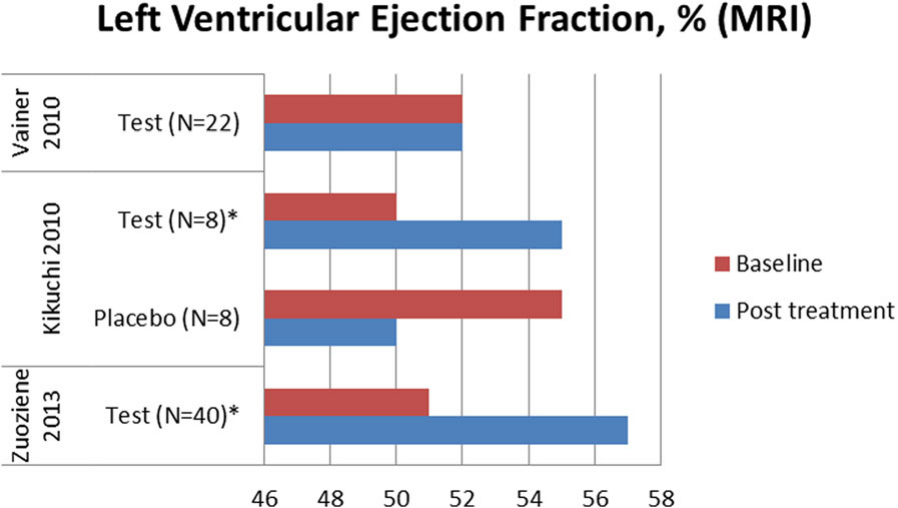
Changes of left ventricular ejection fraction evaluated by magnetic resonance imaging in cardiac shock wave therapy studies. *=*p*<0.05 compared to baseline

**Fig. 6:**
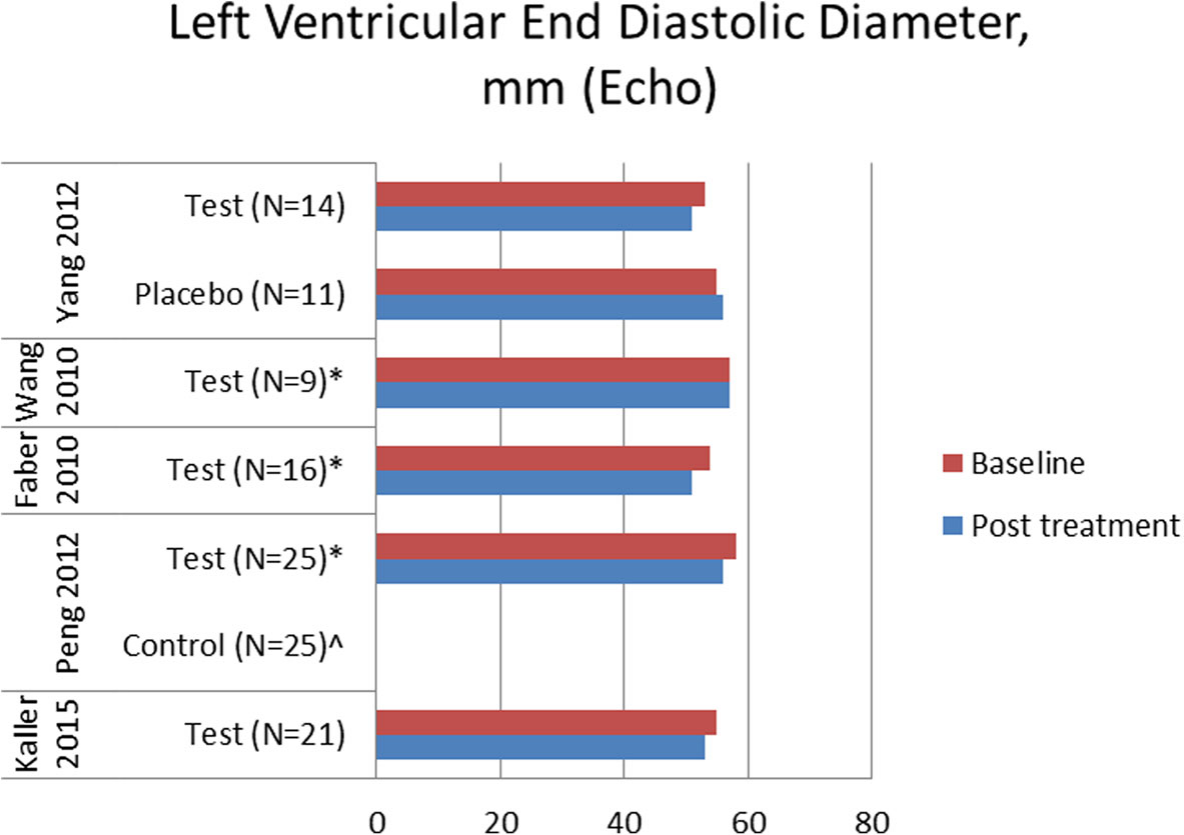
Changes of left ventricular end diastolic diameter in cardiac shock wave therapy studies. *=*p*<0.05 compared to baseline, ^ = no significant changes, no figures indicated

Figures 4 and 5 demonstrate changes of rest left ventricular (LV) function by echocardiography and magnetic resonance imaging (MRI), respectively. Changes of LV end diastolic diameter are shown in Fig. 6. Seven studies demonstrated significant LV function improvement due to CSWT, while in eight studies no statistically significant changes were found.

### Cardiac shock wave therapy effect on myocardial perfusion

During SPECT significant improvement of myocardial perfusion was demonstrated in 27 of 32 studies, during PET in two of four studies. Beneficial changes of myocardial perfusion were associated with increase of LVEF in seven of 13 studies with modest effect of 3.58% (2.0, 4.57). Cassar et al. [27] compared segments that were treated with CSW and those that were not, and found that after 4 months of follow–up the progression of ischemic burden of untreated segments was significantly greater.

### Cardiac shock wave therapy effect on angiogenesis markers

Angiogenesis markers were assessed in four studies. Increased VEGF concentration was revealed after CSWT [28–30]. Kikuchi et al. found that the number of circulating progenitor cells (CD 34^+^/KDR^+^ and CD 34^+^/KDR^+^/c-kit^+^) in peripheral blood remained unchanged [31]. Cai et al. observed significant increase in the number of circulating progenitor cells (CD45^low^/CD34^+^/VEGFR2) in peripheral blood [30].

### Generation of shock waves and cardiac shock wave treatment

Shock waves (SW) belong to acoustic waves that can be transmitted through a liquid medium and focused with a precision of several millimetres to any intended treatment area inside the body.

**Fig. 7:**
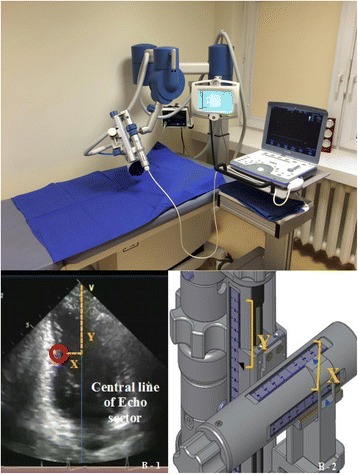
The methodology of cardiac shock wave therapy. **a** Shock wave generator system (Medispec, Germantown, MD, USA) and cardiac imaging system (Vivid i, GE Healthcare, Horten, Norway). **b** Shock wave focal zone alignment: Position of the sub-segment on the 2-dimensional image determined by X and Y coordinates (1). The shockwave applicator position is identically adjusted along X- and Y-axes corresponding to the X and Y coordinates of the ultrasound image (2)

In CAD patients, SW can be delivered to the border of the ischemic area to potentially induce neovascularization from the healthy area to the ischemic zone. Shock waves can be generated by discharge of a high-voltage spark under water or electromagnetic impulse. CSWT is performed using a SW generator system coupled with a cardiac ultrasound imaging system that is traditionally used to target the treatment to area with previously documented ischemia (Fig. 7). SW are delivered via a special applicator through the anatomical acoustic window to the treatment area under electrocardiographic R-wave gating. For optimal therapy, the treatment area is divided into target zones corresponding to the size of the focal zone of the SW applicator (Fig. 7).

## Discussion

Clinical research in intriguing CSWT field continues since 1999, and several new trials are being published every year. The aim of this study was to summarize the results and also to evaluate the quality of currently accumulated evidence on the efficacy of CSWT on CAD treatment. This systematic review expands previously published analysis [32] by including 23 recent studies, and confirms the beneficial effects of CSWT in a larger pooled sample size of patients with stable CAD. The strength of this paper is a systematic character of review, an inclusion in meta-analysis studies with single clinical indication and a uniform treatment protocol, and assessment of bias risk in randomised trials.

In contrast to our study, recently published meta-analysis of Wang and co-authors covered only a limited period of publications, from 2010 to 2014, and included not only English but also Chinese articles [33]. As a result, our work presents the largest contemporary review of human CSWT trials incorporating all the research period.

Like in the previous analyses the majority of detected trials are relatively small, single centre, single arm, some of them insufficiently report methodology and results. In order to avoid substantial heterogeneity and publication bias reported by Wang, we excluded from meta-analysis studies, which targeted at different population of ischemic heart failure, and also non-English articles as potentially producing more beneficial results. Our study focused on the stable CAD patients and confirmed consistent positive anti-anginal effect of CSWT.

In medical field high-energy extracorporeal shock wave therapy (ESWT) was introduced more than 30 years ago as a treatment option for urinary tract stones [34]. ESWT has changed the treatment of urinary calculi, and even today it remains the primary treatment in most non-complicated cases [35]. ESWT has also been applied in biliary tract [36], pancreatic [37] and salivary stones treatment [38]. Low energy ESWT has regenerative features and has been developed as a treatment standard for a variety of orthopedic and soft tissue diseases [39], including wound healing in diabetic patients [40]. Furthermore, shockwaves have been used for treatment chronic pelvic pain syndrome [41] and erectile dysfunction. The observed immediate increase in blood flow due to local vasodilatation and the formation of new capillaries in the treated tissue [16, 17] has led to one of its more promising application in cardiovascular medicine as a possible treatment for patients with stable angina.

The mechanism of CSWT action is multifactorial. SW induces tissue cavitation, leading to a variety of biochemical effects, including shear stress on cell membranes [42], an increase in nitric oxide synthesis [43–46], an up-regulation of vascular endothelial growth factor (VEGF), [14], acceleration of bone marrow cell differentiation into endothelial cells [47], an increase of the amount of circulating endothelial progenitor cells [15]. Thus, CSWT may enhance angiogenesis, reduce inflammatory response, oxidative stress, cellular apoptosis and fibrosis [14, 47, 48]. It is presumed that these mechanisms demonstrated in experimental settings could be translated into clinical effects of improvement of symptoms and myocardial perfusion in CAD patients.

Our review and meta-analysis show that in the majority of published CSWT studies, nitroglycerine consumption and angina frequency decreases, CCS, SAQ scores and NYHA class improves, myocardial perfusion and exercise capacity increases significantly. Most benefits could be observed as early as in the first month, suggesting the contribution of an early local vasodilating effect of SW. Those beneficial effects persisted during the 1-year of follow up, probably related to angiogenesis and other tissue reactions [49, 50].

Total exercise capacity is one of the most important variables used to assess efficacy of any anti-anginal treatment. We evaluated data from randomized clinical studies along with several non-controlled studies of good quality, though certain extent of heterogeneity is not avoided. Our meta-analysis of 596 participants suggests at least a moderate improving effect of CSWT on exercise tolerance.

However, most of the studies included in the review and meta-analysis are single-centre and uncontrolled, making the likelihood of bias towards larger intervention effect substantial. Different methodological quality, inadequate design or unbalanced analysis compels cautious interpretation of the real CSWT effect. Moreover, Wang assessment of methodology confirms our findings that quality of published controlled trials methodology was low [33]. The majority of the randomised studies were evaluated as having high risk if bias in terms of attribution, sample size calculation, blinding of participants and outcome assessment.

Despite very well tolerance, virtually absence of side effects, considerable symptomatic effect and non-invasive nature of CSWT it has not been widely put into practice. This may be associated with the need of special average cost equipment, particular skills of ultrasound scanning and CSWT application, and with the significant time consumption for the whole therapy course as well. Therefore, CSWT can be considered not as a substitutive but as adjunct therapy in case of limited efficacy of optimal medical treatment.

It seems that the tentative phase of this novel treatment lasted enough, and still there is a lack of high quality evidence. This warrants to perform adequately powered double blind, randomized, placebo controlled study in patients with CAD. Currently appropriately designed multicentre study is ongoing with the aim to confirm the additional improvement of exercise tolerance due to CSWT (NCT02339454).

## Conclusions

Systematic review of CSWT studies in stable CAD demonstrated a clinically significant improvement of clinical variables including angina class and quality of life, as well as positive changes in LV function and perfusion. Meta-analysis showed moderate improvement in exercise capacity. Overall, CSWT is a potentially effective new non-invasive option for patients with CAD, but evidence is limited to small low/moderate quality single-centre studies. Multicentre adequately powered randomised double blind studies are warranted.

## Abbreviations

CAD: Coronary artery disease
CCS: Canadian Cardiology Society
CI: Confidence interval
CSWT: Cardiac shock wave therapy
LV: Left ventricular
LVEF: Left ventricular ejection fraction
MRI: Magnetic resonance imaging
NYHA: New York Heart Association
OMT: Optimal medical treatment
PET: Positron emission tomography
RFA: Refractory angina
SAQ: Seattle Angina Questionnaire
SMD: Standardized mean difference
SPECT: Single photon emission computed tomography
SW: Shock waves
VEGF: Vascular endothelial growth factor
